# Trends in Neonatal Intensive Care Unit Utilization in a Large Integrated Health Care System

**DOI:** 10.1001/jamanetworkopen.2020.5239

**Published:** 2020-06-18

**Authors:** David Braun, Eric Braun, Vicki Chiu, Anthony E. Burgos, Mandhir Gupta, Marianna Volodarskiy, Darios Getahun

**Affiliations:** 1 Department of Research and Evaluation, Kaiser Permanente Southern California, Pasadena, California; 2 Women’s and Children’s Health Care Leadership Team, Kaiser Permanente Southern California, Pasadena, California; 3 Department of Pediatrics, Kaiser Permanente Southern California, Panorama City, California; 4 Department of Consulting and Implementation, Kaiser Permanente Southern California, Pasadena, California; 5 Department of Pediatrics, Kaiser Permanente Downey Medical Center, Downey, California; 6 Department of Pediatrics, Kaiser Permanente Bernard J. Tyson School of Medicine, Pasadena, California; 7 Department of Patient Care Services, Kaiser Permanente Southern California, Pasadena, California; 8 Department of Health Systems Science, Kaiser Permanente Bernard J. Tyson School of Medicine, Pasadena, California; 9 Department of Obstetrics and Gynecology, Rutgers-Robert Wood Johnson Medical School, New Brunswick, New Jersey

## Abstract

**Importance:**

There are few population-based studies addressing trends in neonatal intensive care unit (NICU) admission and NICU patient-days, especially in the subpopulation that, by gestational age (GA) and birth weight (BW), might otherwise be able to stay in the room with their mothers.

**Objective:**

To describe population-based trends in NICU admissions, NICU patient-days, readmissions, and mortality in the birth population of a large integrated health care system.

**Design, Setting, and Participants:**

This cohort study was conducted using data extracted from electronic medical records at Kaiser Permanente Southern California (KPSC) health care system. Participants included all women who gave birth at KPSC hospitals and their newborns from January 1, 2010, through December 31, 2018. Data extraction was limited to data entry fields whose contents were either numbers or fixed categorical choices. Rates of NICU admission, NICU patient-days, readmission rates, and mortality rates were measured in the total population, in newborns with GA 35 weeks or greater and BW 2000 g or more (high GA and BW group), and in the remaining newborns (low GA and BW group). Admissions to the NICU and NICU patient-days were risk adjusted with a machine learning model based on demographic and clinical characteristics before NICU admission. Changes in the trends were assessed with 2-sided correlated seasonal Mann-Kendall test. Data analysis was performed in August 2019.

**Exposures:**

Admission to the NICU and NICU patient-days among the birth cohort.

**Main Outcomes and Measures:**

The primary outcomes were NICU admission and NICU patient-days in the total neonatal population and GA and BW subgroups. The secondary outcomes were readmission and mortality rates.

**Results:**

Over the study period there were 320 340 births (mean [SD] age of mothers, 30.1 [5.7] years; mean [SD] gestational age, 38.6 [1.97] weeks; mean [SD] birth weight, 3302 [573] g). The risk-adjusted NICU admission rate decreased from a mean of 14.5% (95% CI, 14.2%-14.7%) to 10.9% (95% CI, 10.7%-11.7%) (*P* for trend = .002); 92% of the change was associated with changes in the care of newborns in the high GA and BW group. The number of risk-adjusted NICU patient-days per birth decreased from a mean of 1.50 patient-days (95% CI, 1.43-1.54 patient-days) to 1.40 patient-days (95% CI, 1.36-1.48 patient-days) (*P* for trend = .03); 70% of the change was associated with newborns in the high GA and BW group. The unadjusted 30-day readmission rates and mortality rates did not change.

**Conclusions and Relevance:**

Admission rates to the NICU and numbers of NICU patient-days decreased over the study period without an increase in readmissions or mortality. The observed decrease was associated with the high GA and BW newborn population. How much of this decrease is attributable to intercurrent health care systemwide quality improvement initiatives would require further investigation. The remaining unexplained variation suggests that further changes are also possible.

## Key Points

**Question:**

How are neonatal intensive care unit (NICU) admission rates and NICU patient-days changing over time for various birth weight, gestational age, and acuity subgroups?

**Findings:**

In this cohort study of neonates in a large integrated health care system, the risk-adjusted NICU admission rate and NICU patient-days decreased from 2010 through 2018 without an increase in readmission or mortality rates. The decrease was associated with the high gestational age and birth weight subgroup.

**Meaning:**

These findings suggest that substantial decreases in NICU utilization across large birth populations are possible, and the remaining unexplained variation suggests that further changes are also possible.

## Teaser

This cohort study describes population-based trends in neonatal intensive care unit admissions and patient-days, readmissions, and mortality rates in the birth population of a large integrated health care system.

## Introduction

Neonatal intensive care unit (NICU) care can be life-saving, but it is also expensive,^1^ common,^2^ and can cause harm.^3,4,5,6,7^ There is wide variation in NICU admission rates and NICU patient-days, especially among lower acuity newborns, suggesting that there are opportunities to optimize the use of NICU care.^2,8,9,10,11^ The value and quality equation for NICU care has been a topic of increasing interest.^10,12^ Stewardship of NICU care itself has been suggested as a choosing wisely area of focus.^11^

To assess NICU care, one needs to study the whole population at risk for NICU admission: both newborns who are admitted to the NICU and those who only require rooming-in care with their mothers. This is especially true for the 98% of births at gestational age 34 weeks or older and the 80% of NICU admissions that do not meet high acuity criteria.^2,11^

There are few population-based studies in this area.^8,9,10,13^ The studies vary in time period of study, choice of birth subpopulations, definition of NICU admissions, and NICU patient-days. Although the overall rate of NICU admissions increased in the last few decades as modern NICU care became established,^10^ we are aware of only 1 recent study^14^ that examined temporal trends in NICU admissions.

The Kaiser Permanente Southern California (KPSC) health care system provides integrated health care for a large (4.7 million), socioeconomically diverse population that is broadly representative of the Southern California population.^15^ All patient care documentation and records are contained within a single electronic health record (EHR) system. This allowed us to look at the whole birth population regardless of patient acuity or birth hospital acuity level. It also allowed us to obtain maternal and neonatal data directly from the EHRs, rather than abstracted data such as coding or claims data. We also could identify patients in the NICU according to care physically occurring in the NICU, rather than claims or birth certificate proxies for NICU care. Finally, KPSC was also in the midst of a number of systemwide quality improvement initiatives expected to decrease NICU admissions and patient-days. The aims of this study were to describe the contribution to NICU admissions and NICU patient-days by several subpopulations of interest, temporal trends in rate of NICU admission and NICU patient-days, a risk adjustment model controlling for the contribution of pre-NICU risk factors, and readmission and mortality balancing factors.

## Methods

For this retrospective cohort study of NICU utilization, we extracted data from the EHR on neonates born in 13 of 14 KPSC facilities from January 1, 2010, through December 31, 2018, regardless of membership status at the time of NICU stay. This study was approved by the KPSC institutional review board with exemption of the requirement to obtain informed consent because the data were deidentified.

One of the 14 hospitals was excluded from this study, because this level I nursery did not have a formal NICU. Furthermore, we excluded member mothers whose newborns were delivered at non-KPSC (contracting) hospitals. Information stored within the EHR includes demographic data, including maternal race/ethnicity, age, education, medical insurance type, address, all inpatient and outpatient care, linkage of individual newborns’ and mothers’ charts, and care interventions entered at the time they occurred often in number- or list-delimited fields. California Perinatal Quality Care Collaborative registry data on the population was downloaded from the California Perinatal Quality Care Collaborative website.^16^

Care in the NICU was defined as care physically occurring in a NICU bed on the basis of coding of the bed to a NICU cost center. The NICU admission rate was defined as the frequency of neonates born at KPSC hospitals who received NICU care between birth and discharge to home or mortality. The NICU patient-days were determined by the sum of patient-hours assigned to NICU cost center across the birth admission and any stay associated with subsequent intrahospital or interhospital NICU transfers. Intrahospital or interhospital NICU transfer was defined as a new admission to another medical center within 24 hours where the previous admission’s discharge order did not indicate discharge to home. Information on patient-days for NICU services provided in contracting hospitals was obtained from insurance claims. Readmission was defined as a NICU admission to any KPSC hospital occurring 24 hours or more after the previous discharge or an admission occurring less than 24 hours after previous discharge with documented discharge order indicated as discharge to home or if the new admission was to the same medical center. In this study, mortality was defined as death from the time of birth to 30 days after discharge from NICU. Neonates in the high GA and BW group were defined as having gestational age 35 weeks or older and birth weight 2000 g or higher. Low GA and BW newborns were defined as those with gestational age less than 35 weeks or birth weight less than 2000 g. High acuity newborns were defined as patients meeting 1 or more California Perinatal Quality Care Collaborative high acuity inclusion criteria: birth weight of 401 to 1500 g, gestational age between 22^0/7^ and 31^6/7^ weeks, death, invasive or noninvasive assisted ventilation for 4 hours or more, early bacterial sepsis, major surgical procedure requiring anesthesia, interhospital transport to or from the NICU for higher acuity care, suspected encephalopathy or suspected perinatal asphyxia, and active therapeutic hypothermia.^16^ Newborns who did not meet 1 or more of the high acuity criteria were classified as low acuity. Surgical newborns were defined as newborns who had a completed surgical procedure recorded in the Electronic Privacy Information Center OpTime surgery log, which is the EHR module used for all surgical procedures. Operations performed outside KPSC facilities were identified through claims data associated with the neonates born in KPSC facilities.

The contribution of high GA and BW trends to the total population trends was calculated as follows: the difference between the 2010 and 2018 rates was calculated for each population. Next, the high GA and BW difference was divided by the total population difference, weighted by the total number of newborns in each population.

### Risk Adjustment Model

This study was designed to assess the association of postnatal care practices with neonatal outcomes while controlling for independent factors. For this reason, we developed an adjustment model focused on socioeconomic, prenatal, and delivery room variables that have been extracted from the maternal and newborn records that might affect or describe patient acuity risk independent of postnatal care management. LightGBM,^17^ a machine learning model, was used to model NICU admission and NICU patient-days. LightGBM has outstanding predictive performance while accommodating issues such as complex interactions of candidate factors, nonlinearity, and missing data.^17^ A detailed description of the risk adjustment model variables is presented in the eAppendix in the Supplement.

### Statistical Analysis

We assessed differences in the distribution of maternal characteristics using the χ^2^ test for categorical variables and *t* test for continuous variables using SAS statistical software version 9.4 (SAS Institute). We performed trend analyses of monthly NICU admissions per live birth, NICU patient-days, readmissions, and mortality per 100 live births. The analyses tested whether these metrics had statistically significant trends over time. The trend analysis included assessments of both crude and risk-adjusted trends for the NICU utilization metrics and only crude trends for the readmission and mortality metrics. Confidence limits for risk adjusted trends were calculated using bias-corrected bootstrapping performed with Python statistical software version 3.7.4 and Python Package SciPy version 1.3.1.18 (both from Python Software Foundation). Confidence limits for readmissions and mortality were calculated using Agresti-Coull confidence intervals for binomial proportions^18^ using Python statistical software version 3.7.7 and Python Package statsmodels version 0.11.1 (both from Python Software Foundation). The trend analyses were performed with 2-sided correlated seasonal Mann-Kendall tests calculated using Python statistical software version 3.7.4 and Python Package PyMannKendall version 1.2 (both from Python Software Foundation).^19^ *P* < .05 was considered statistically significant. Monthly means were used to ensure the analyses had sufficient power. Data analysis was performed in August 2019.

Systemwide quality improvement initiatives over the study period included decreasing obligate NICU admission guidelines for well-appearing newborns to GA less than 35 weeks and BW less than 2000 g (implementation in October 2014), use of a newborn early-onset sepsis risk calculator for decision support^20^ (implementation in October 2013), minimization of elective deliveries at GA less than 39 weeks^21,22^ (implementation in March 2014), and maximization of vaginal delivery for nulliparous, term, singleton, vertex pregnancies (implementation in May 2017).^23^ Each of these initiatives has been associated with decreasing the need for NICU care.^21,23,24,25^

## Results

 A total of 320 340 eligible women (mean [SD] age, 30.1 [5.7] years) gave birth during the study period, representing 16% of births in Southern California. Neonates had a mean (SD) gestational age of 38.6 (1.97) weeks and a mean (SD) birth weight of 3302 (573) g. Approximately 10% of KPSC members who were pregnant did not receive their birth hospitalization care in KPSC hospitals or gave birth at the 1 KPSC hospital without a NICU. Compared with mothers of newborns not admitted to the NICU, mothers of newborns admitted to the NICU were more likely to be older (aged ≥35 years, 63 080 mothers [22.44%] vs 10 354 mothers [26.40%]), black (23 014 mothers [8.19%] vs 4707 mothers [12.00%]), nulliparous (56 466 mothers [20.09%] vs 10 823 mothers [27.60%]), have lower household income (median annual household income $30 000-$49 999, 75 348 mothers [26.80%] vs 10 994 mothers [28.03%]), non-Medicaid recipients (253 904 mothers [90.32%] vs 35 682 mothers [90.98%]; overall, 9.60% of the KPSC population is insured by Medicaid), and with multiple gestation (5743 mothers [2.04%] vs 5169 mothers [13.18%]) (Table 1). Neonates admitted to the NICU were more likely than those not admitted to be male (21 577 newborns [55.02%] vs 142 079 newborns [50.54%]), of low GA (22-29 weeks, 2332 newborns [5.95%] vs 136 newborns [0.05%]), and low BW (501-750 g, 560 newborns [1.43%] vs 101 newborns [0.04%]).

**Table 1.** Maternal Characteristics by NICU Admission Status

Characteristics	Mothers, No. (%)		
	Total live births (N = 320 340)	NICU admission status^a^	
		Not admitted (n = 281 120)	Admitted (n = 39 220)
Maternal age, y			
<20	11 061 (3.45)	9670 (3.44)	1391 (3.55)
20-29	131 025 (40.9)	116 059 (41.28)	14 966 (38.16)
30-34	104 815 (32.72)	92 306 (32.84)	12 509 (31.89)
≥35	73 434 (22.92)	63 080 (22.44)	10 354 (26.40)
Race/ethnicity			
Non-Hispanic white	95 464 (29.80)	83 776 (29.80)	11 688 (29.80)
Non-Hispanic black	27 721 (8.65)	23 014 (8.19)	4707 (12.00)
Hispanic	141 353 (44.13)	125 111 (44.50)	16 242 (41.41)
Asian or Pacific Islander	46 603 (14.55)	41 045 (14.60)	5558 (14.17)
Other	7151 (2.23)	6338 (2.25)	813 (2.07)
Unknown	2048 (0.64)	1836 (0.65)	212 (0.54)
Neonate’s sex			
Female	156 648 (48.90)	139 015 (49.45)	17 633 (44.96)
Male	163 656 (51.09)	142 079 (50.54)	21 577 (55.02)
Annual median household income, $			
<30 000	13 853 (4.32)	12 026 (4.28)	1827 (4.66)
30 000-49 999	86 342 (26.95)	75 348 (26.80)	10 994 (28.03)
50 000-69 999	104390 (32.59)	91 868 (32.68)	12 522 (31.93)
70 000-89 999	59 172 (18.47)	51 990 (18.49)	7182 (18.31)
≥90 000	49 928 (15.59)	44 186 (15.72)	5742 (14.64)
Parity			
Multipara	221 231 (69.06)	197 048 (70.09)	24 183 (61.66)
Nullipara	67 289 (21.01)	56 466 (20.09)	10 823 (27.60)
Plurality			
Singleton	309 427 (96.59)	275 376 (97.96)	34 051 (86.82)
Multiple	10 912 (3.41)	5743 (2.04)	5169 (13.18)
Medicaid insurance			
No	289 586 (90.40)	253 904 (90.32)	35 682 (90.98)
Yes	30 754 (9.60)	27 216 (9.68)	3538 (9.02)
Gestational age, wk			
22-29	2468 (0.77)	136 (0.05)	2332 (5.95)
30-33	5101 (1.59)	53 (0.02)	5048 (12.87)
34	4095 (1.28)	247 (0.09)	3848 (9.81)
35	6032 (1.88)	2676 (0.95)	3356 (8.56)
36	12 169 (3.80)	9093 (3.23)	3076 (7.84)
37-38	81 632 (25.48)	74 292 (26.43)	7340 (18.71)
39-40	181 864 (56.77)	169 934 (60.45)	11 930 (30.42)
≥41	26 979 (8.42)	24 689 (8.78)	2290 (5.84)
Birth weight, g			
401-500	10 (0.00)	5 (0.00)	5 (0.01)
501-750	661 (0.21)	101 (0.04)	560 (1.43)
751-1000	798 (0.25)	15 (0.01)	783 (2.00)
1001-1250	919 (0.29)	10 (0.00)	909 (2.32)
1251-1500	1320 (0.41)	14 (0.00)	1306 (3.33)
1501-1750	1744 (0.54)	32 (0.01)	1712 (4.37)
1751-1999	2771 (0.87)	201 (0.07)	2570 (6.55)
2000-2499	14 287 (4.46)	7463 (2.65)	6824 (17.40)
2500-3999	269 788 (84.22)	248 232 (88.30)	21 556 (54.96)
4000-4499	24 092 (7.52)	21 798 (7.75)	2294 (5.85)
≥4500	3948 (1.23)	3249 (1.16)	699 (1.78)
Year of NICU admission			
2010	29 806 (9.30)	25 083 (8.92)	4723 (12.04)
2011	31 891 (9.96)	27 015 (9.61)	4876 (12.43)
2012	33 623 (10.5)	29 036 (10.33)	4587 (11.70)
2013	34 004 (10.61)	29 723 (10.57)	4281 (10.92)
2014	35 216 (10.99)	30 989 (11.02)	4227 (10.78)
2015	36 766 (11.48)	32 664 (11.62)	4102 (10.46)
2016	39 099 (12.21)	34 812 (12.38)	4287 (10.93)
2017	39 660 (12.38)	35 519 (12.63)	4141 (10.56)
2018	40 275 (12.57)	36 279 (12.91)	3996 (10.19)

Abbreviation: NICU, neonatal intensive care unit.

^a^ Differences between NICU admitted and not admitted by maternal and child characteristics were statistically significant (*P* < .001).

Among the live births during the study period, 39 220 newborns (12.24%) were admitted to the NICU, generating a mean of 1.46 NICU patient-days per birth (Table 2). High GA and BW newborns accounted for 96% (307 417) of births, 69% (26 918) of NICU admissions, and 25% (118 027) of NICU patient-days. Admissions to the NICU and NICU patient-days were also enumerated for other subpopulations, such as very low birth weight newborns, surgical newborns, high acuity newborns, and newborns at various gestational ages.

**Table 2.** Subpopulation of NICU Utilization, 2010-2018

Acuity group	Neonates, No.	Neonates, No. (%)					
		NICU admission per total		NICU patient-days per total		Readmission <30 d after discharge per total live births (n = 320 340)	Mortality ≤30 d after discharge per total live births (n = 320 340)
		Live births (n = 320 340)	NICU admissions (n = 39 220)	Live births (n = 320 340)	NICU patient-days (n = 468 236)		
Total population	320 340	39 220 (12.24)	39 220 (100.00)	468 236 (1.46)	468 236 (100.00)	7663 (2.39)	895 (0.28)
High GA (≥35 wk) and BW (≥2000 g)	307 417	26 918 (8.40)	26 918 (68.63)	118 027 (0.37)	118 027 (25.21)	7180 (2.24)	326 (0.10)
Low GA (<35 wk) and BW (<2000 g)	12 923	12 302 (3.84)	12 302 (31.37)	350 208 (1.09)	350 208 (74.79)	483 (0.15)	569 (0.18)
High acuity newborns							
All CPQCC newborns	10 155	9735 (3.04)	9735 (24.82)	320 317 (1.00)	320 317 (68.41)	623 (0.19)	684 (0.21)
Small (BW≤1500 g) CPQCC newborns with GA 22-31^6/7^ wk	4462	4327 (1.35)	4327 (11.03)	233 874 (0.73)	233 874 (49.95)	248 (0.08)	423 (0.13)
Large (BW>1500 g) CPQCC newborns with GA≥32 wk	5693	5408 (1.69)	5408 (13.79)	86 443 (0.27)	86 443 (18.46)	375 (0.12)	261 (0.08)
High acuity surgical CPQCC newborns	1561	1558 (0.49)	1558 (3.97)	93 840 (0.29)	93 840 (20.04)	178 (0.06)	95 (0.03)
All newborns with GA≥34 weeks	312 771	31 840 (9.94)	31 840 (81.18)	175 580 (0.55)	175 580 (37.50)	7319 (2.29)	419 (0.13)
Low acuity newborns (GA≥34 wk; non-CPQCC, and BW>1500 g)	307 817	27 165 (8.48)	27 165 (69.26)	105 492 (0.33)	105 492 (22.53)	6979 (2.18)	179 (0.06)
With NICU LOS<3 d	15 977	15 972 (4.99)	15 972 (40.72)	18 488 (0.06)	18 488 (3.95)	401 (0.13)	4 (0.00)
With NICU LOS 3 d	11 203	11 196 (3.50)	11 196 (28.55)	87 059 (0.27)	87 059 (18.59)	257 (0.08)	7 (0.00)
Moderately preterm born GA 30-33^6/7^ wk, total	5101	5048 (1.58)	5048 (12.87)	130 552 (0.41)	130 552 (27.88)	189 (0.06)	89 (0.03)
Late preterm born GA 34-35^6/7^ wk	10 127	10 280 (2.25)	10 280 (18.37)	90 103 (0.22)	90 103 (15.04)	873 (0.11)	172 (0.03)

Abbreviations: BW, birth weight; CPQCC, California Perinatal Quality Care Collaborative; GA, gestational age; LOS, length of stay; NICU, neonatal intensive care unit.

The overall readmission rate in the 30 days after discharge was 2.39% (Table 2). The overall mortality rate between birth and 30 days after discharge was 0.28%.

The NICU admission model adjusted for demographic, antenatal, and delivery room risk factors had an area under the receiver operator curve of (0.895) for the total population and (0.849) for newborns with high GA and BW. The mean precision (ie, area under the precision-recall curve) was (0.740) for the total population and (0.529) for newborns with high GA and BW. The NICU patient-days risk-adjustment model had an *R*^2^ of 0.729 and a root mean squared error of 10.712 for the total population and an *R*^2^ of 0.233 and a root mean squared error of 7.312 days for the high GA and BW newborns.

**Figure 1.**
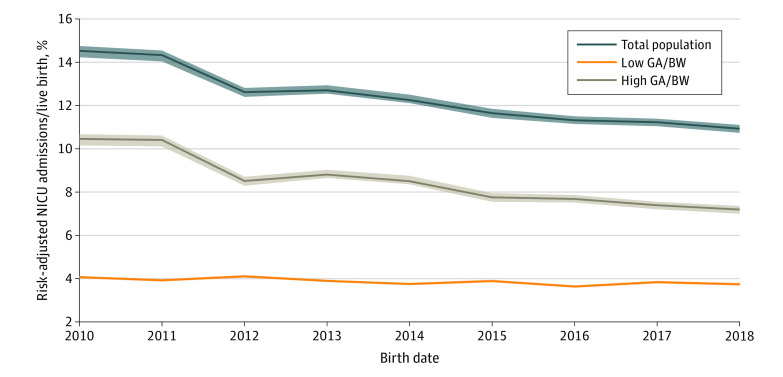
Trends in Risk-Adjusted Neonatal Intensive Care Unit (NICU) Admissions per Live Birth, 2010-2018 Risk adjustments were made for maternal and newborn socioeconomic, prenatal, and delivery room variables. Lines denote means, and shaded areas denote 95% confidence limits. High gestational age (GA) is 35 weeks or more, and high birth weight (BW) is 2000 g or more. Low GA is less than 35 weeks, and low BW is less than 2000 g. Statistical significance was assessed with Mann-Kendall tests.

**Figure 2.**
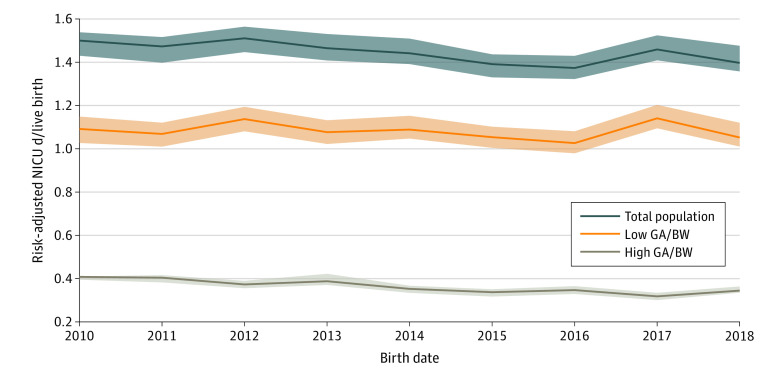
Trends in Risk-Adjusted Neonatal Intensive Care Unit (NICU) Patient-Days Per Live Birth, 2010-2018 Risk adjustments were made for maternal and newborn socioeconomic, prenatal, and delivery room variables. Lines denote means, and shaded areas denote 95% confidence limits. High gestational age (GA) is 35 weeks or more, and high birth weight (BW) is 2000 g or more. Low GA is less than 35 weeks, and low BW is less than 2000 g. Statistical significance was assessed with Mann-Kendall tests.

Over the study period the risk-adjusted total population NICU admission rate decreased from a mean of 14.5% (95% CI, 14.2%-14.7%) of births to 10.9% (95% CI, 10.7%-11.7%) of births, a 25% relative decrease (*P* for trend = .002) (Figure 1). The NICU admission rate for the high GA and BW subgroup decreased from a mean of 10.5% (95% CI, 10.2%-10.7%) of births to 7.2% (95% CI, 7.0%-7.4%) of births (*P* for trend = .002), accounting for 92% of the total decrease. The NICU admission rate for the low GA and BW subgroup decreased from a mean of 4.1% (95% CI, 4.0%-4.1%) of births to 3.7% (95% CI, 3.7%-3.8%) of births (*P* for trend = .005). Over the study period, the risk-adjusted total NICU patient-days per birth decreased from a mean of 1.50 patient-days (95% CI, 1.43-1.54 patient-days) to 1.40 patient-days (95% CI, 1.36-1.48 patient-days) (*P* for trend = .03). This represented a 7% relative decrease; 70% of the change was associated with newborns in the high GA and BW group (Figure 2). The risk-adjusted total NICU patient-days per birth for the high GA and BW subgroup decreased from a mean of 0.41 patient-days (95% CI, 0.40-0.41 patient-days) to 0.34 patient-days (95% CI, 0.34-0.36 patient-days) (*P* for trend = .003). The decrease in NICU patient-days per birth for the low GA and BW subgroup from 1.09 patient-days (95% CI, 1.03-1.15 patient-days) to 1.05 patient-days (1.01-1.12 patient-days) was not statistically significant (*P* for trend = .88).

There were no statistically significant trends in 30-day readmission rates during the study period. The total birth population’s 30-day readmission rates mean decrease from 2.7% (95% CI, 2.5%-2.9%) to 2.1% (95% CI, 1.9%-2.3%) was not statistically significant (*P* for trend = .12). The High GA and BW subgroup decrease from 2.6% (95% CI, 2.3%-2.7%) to 2.0% (95% CI, 1.8%-2.1%) of births (*P* for trend = .10) and low GA and BW decrease from 0.2% (95% CI, 0.2%-0.3%) to 0.1% (95% CI, 0.1%-0.2%) of births (*P* for trend = .62) were also not statistically significant.

There were no statistically significant trends in mortality rates during the period. The total birth population’s mortality rate from birth to 30 days after discharge to home ranged from 0.25% (95% CI, 0.20%-0.32%) to 0.32% (95% CI, 0.26%-0.37%) (*P* for trend = .88). The rate for the high GA and BW subgroup ranged from 0.08% (95% CI, 0.07%-0.14%) to 0.14% (95% CI, 0.10%-0.17%) (*P* for trend = .79). The rate for the low GA and BW subgroup ranged from 0.16% (95% CI, 0.11%-0.20%) to 0.20% (95% CI, 0.14%-0.22%) (*P* for trend = .87).

## Discussion

This retrospective cohort analysis of a large integrated health care system provides population-based, risk-adjusted estimates of NICU admission and NICU patient-day demand for clinically important subpopulations based on data extracted directly from the medical records. The findings suggested a 25% relative decrease in NICU admissions during the study period, from 14.5% of births in 2010 to 10.9% of births in 2018. This trend is contrary to that found by Harrison et al,^14^ the only other population-based study on trends in NICU admissions, which showed a 23% increase NICU utilization from 6.4% in 2007 to 7.8% in 2012. The rates found by Harrison et al,^14^ were only approximately one-half the rates reported in other studies,^2,26,27^ perhaps because of the limitation of birth certificate data^28,29^ and the limitation to centers with high acuity NICUs, which represent only a minority of delivery centers.^9^

Recently published population-based cross-sectional studies showed notable interhospital variation in permutations of NICU admission rates, NICU patient-days, and other measures of utilization, especially in the lower acuity patients, such as newborns with high GA and BW. First, in a study^2^ of California Perinatal Quality Care Collaborative newborns in 2015, the overall NICU admission rate based on hospital self-reports was 12%; among the 358 453 neonates born at GA 34 weeks or later, the rate was 10%. Second, in a study^10^ of US newborns between 2010 and 2013, the NICU admission rate based on Anthem insurance claims was 6.2% among 1 110 517 low-risk newborns and 96.8% among 12 086 very low birth weight newborns. Third, in a study^26^ of Texas Medicaid births between 2010 and 2014, the NICU admission rate was 11.6% and NICU patient-days were 2.0 days per birth among 965 201 births. The overall NICU admission rate was 37.5% among 78 013 singleton births at GA 34 to 36^6/7^ weeks (late preterm) and 14.9 days per late preterm birth among 37 460 singleton late preterm births.^26^ Fourth, in Norway between 2009 and 2014, the overall NICU admission rate was 9.4% among 368 068 births, 45% in among births at GA 34^0/7^ to 36^6/7^ weeks, and 6% for GA 37 weeks or later, with a mean of 5.3 NICU patient-days for GA 37 weeks or later, 11.3 NICU patient-days for GA 34^0/7^ to 36^6/7^ weeks, and 40 NICU patient-days for GA less than 34 weeks.^27^ Finally, a March of Dimes study^13^ from July 2009 to June 2010 of 183 030 births from hospitals contributing data to the National Perinatal Information Center Quality Analytic Services reported an overall NICU admission rate of 14.4% and 1.9 NICU patient-days per birth. In that study,^13^ the NICU admissions included neonates admitted from referring hospitals, which may have resulted in an overestimate of NICU admissions and NICU patient-days arising from the reporting hospitals’ birth populations. In the present study, the mean NICU admission rate of 12.24% was similar to those in 2 US studies^2,26^ but higher than that in the Norwegian study (9.8%).^27^ By the end of our study period, our NICU admission rate of 10.9% was approximately one-half the difference between the Norwegian and US studies.

There was also a 7% relative decrease in NICU patient-days, from 1.50 to 1.40 days per birth, during the study period. The smaller change in NICU patient-days compared with NICU admissions is consistent with the small proportion of total NICU patient-days contributed by the high GA and BW newborn admissions. These rates were lower than those in the US studies,^13,26^ where overall NICU patient-days ranged from 1.9 to 2.0 NICU patient-days per birth, although they were higher than 1.2 NICU patient-days per birth reported from Norway.^27^ Although these differences appear small, they are associated with a very large number of actual NICU patient-days. For instance, the US has approximately 4 million births per year^30^; thus, a change of 0.1 NICU patient-day per birth would generate 400 000 NICU patient-days per year.

The decreases in our NICU admissions and NICU patient-days were not associated with increases in readmission rates and mortality. The readmission^31,32,33^ and mortality^34^ rates compare favorably with published rates. This suggests that the observed temporal decrease in NICU utilization have not been at the cost of these complications.

Our risk adjustment model was designed to reveal changes in postnatal care practices by adjusting for a rich range of clinical factors and excluding any adjustment for care at or after the decision to admit to the NICU. This suggests that the changes in NICU admissions and patient-days we observed were less likely to be associated with differences in case mix and more likely associated with changes in the decision-making around NICU admission and NICU care. The model still shows a significant amount of unexplained variation in both NICU admissions and patient-days. For instance, in the high GA and BW subgroup, the area under the precision-recall curve was 0.529 for NICU admissions and 0.233 for NICU patient-days. Because we controlled for primary patient acuity factors, the unexplained variation is likely associated with heterogeneous care practices rather than the clinical characteristics of the newborns, suggesting that there are still large opportunities for improving care practices in NICU care.

The high GA and BW newborns were associated with the majority of the decrease in total NICU admissions observed in our study. These changes may have been associated with the intercurrent systemwide quality improvement initiatives during the study period that primarily targeted the high GA and BW subgroup.

Our health care system’s financial structure is population based rather than fee-for-service based, so financial incentives are more aligned with decreasing rather than increasing the amount and cost of NICU care. The association of this form of reimbursement with observed NICU care trends may be worth assessing further.

### Strengths and Limitations

This study has multiple strengths. First, the findings of this study can be generalizable to Southern California pregnant women and their newborns because it is based on a large cohort (16% of births in Southern California) that is demographically representative of the Southern California population.^15,35^ Second, the size of the study population was similar to that of studies by Schulman et al^2^ and Moen et al,^27^ approximately one-third the size of the Texas study^26^ and the Anthem study,^10^ but much smaller than the study by Harrison et al.^14^ The BW and GA birth subpopulation distribution was similar to other population-based studies.^2,11,14,26,27^ Third, 9.60% of our member population is Medicaid insured. Furthermore, our analyses adjusted for type of insurance and US Census tract–based socioeconomic characteristics. Fourth, we also were able to use a more direct measure of NICU care based on the daily physical location of the newborn as recorded in the EHR, avoiding errors introduced by other NICU admission proxies, such as self-reports,^2^ insurance claims,^10^ and birth certificate data.^28,29^ Fifth, we included care across interhospital transfer admissions, avoiding underestimates of NICU patient-days from studies relying on birth admission data alone. Sixth, our approach did not require geographical proxies to match births to NICU admissions, avoiding mismatching of births to NICU care. Seventh, we chose the high GA and BW subgroup as a focus because this is a large population and one of low enough endogenous risk for decompensation that, barring specific individual problems, they would be permitted by many physicians to room in with their mother. This made the high GA and BW population particularly likely to benefit from the intercurrent NICU-sparing quality improvement initiatives we enumerated, where NICU admission itself was the major modifiable care decision. In this report, we did not provide data to quantify the absolute or relative effects of these quality improvement initiatives. There are challenges to this work, including identifying quantifiable measures of patient-level compliance and determining to what extent the initiatives complemented or conflicted with the effects of other initiatives. These would be a worthwhile area for future research. In a related way, the remaining unexplained variation in NICU admissions among high GA and BW newborns suggests that there is potential for further optimization of care, possibly through improved compliance with the listed initiatives, as well as the introduction of additional changes in obstetrical or neonatal practice.

This study also has several limitations. First, NICU patient-days were not weighted by nursing or respiratory care acuity, so the extrapolation of changes in NICU patient-days on NICU resources is limited. Second, approximately 10% of KPSC members who were pregnant did not receive their birth hospitalization care in KPSC hospitals or gave birth at the 1 KPSC hospital without an NICU; this raises the possibility of selection bias. Because most of these hospitals are geographically peripheral to the KPSC population, the selection choice of delivery hospital was less likely to be associated with nongeographical factors. We did not do sensitivity analysis to confirm whether there was not a difference in case mix in this population because information allowing for sensitivity analysis for this population was limited.

## Conclusions

We observed a decrease in NICU admissions and patient-days in a large, Southern California–representative population in an integrated health care system. This trend was the opposite of that reported in another population-based study on NICU admissions. Most of the decrease was associated with the decrease in NICU use among the high GA and BW population. It would require further investigation to understand how much of this decrease was attributable to intercurrent health care systemwide quality improvement initiatives. The remaining unexplained variation suggests further changes are also possible.

## Notes

Supplement. **eAppendix.** Risk Adjustment Model Variables **eReferences**
