# Supplementary material for: Trends in Neonatal Intensive Care Unit Utilization in a Large Integrated Health Care System
Source: JAMA Netw Open. 2020 Jun 18;3(6):e205239. doi: 10.1001/jamanetworkopen.2020.5239 (PMC7303809; doi:10.1001/jamanetworkopen.2020.5239)
Supplement: Supplement. — eAppendix. Risk Adjustment Model Variables eReferences [file jamanetwopen-3-e205239-s001.pdf]

Supplementary Online Content

Braun D, Braun E, Chiu V, et al. Trends in neonatal intensive care unit utilization in a large integrated health care system. *JAMA Netw Open*. 2020;3(6):e205239. doi:10.1001/jamanetworkopen.2020.5239

eAppendix. Risk Adjustment Model Variables

eReferences

This supplementary material has been provided by the authors to give readers additional information about their work.

© 2020 Braun D et al. *JAMA Network Open.*

eAppendix: Risk Adjustment Model Variables

Adjustment model variables included: maternal age; race/ethnicity (non-Hispanic White [White], non-Hispanic Black [Black], Hispanic, and Asian/Pacific Islander and “other race”; American Community Survey census tract level educational attainment (elementary, high school, college, and graduate), median family household income, unemployment rate, and population density by geocoded maternal residence[1]; self-reported smoking during pregnancy (yes/no); infant’s sex; type of gestation (singleton or multiple); gravidity; parity; timing of prenatal care initiation (early or first trimester and no or initiated after the first trimester); magnesium sulfate given within 3 months of delivery admission; steroid treatment within 3 months of delivery admission; delivery methods (vaginal or cesarean); cesarean section intervention by degree of obstetrical concern for the fetus, described by the cesarean delivery indication text field grouped as follows from least to greatest concern: 1) previous c-section, VBAC not indicated, declined VBAC, primary elective C-Section, placenta accreta, prematurity, HSV; 2) Other Medical Necessity, multiple gestation, prematurity, preeclampsia/eclampsia, previa, breech, malpresentation other than breech, failed Induction, failed instrumentation, failure to progress first stage labor, failure to progress second stage labor; 3) fetal intolerance to labor, placental abruption, prolapsed cord. gestational age at birth (≥20 completed weeks); birth weight in grams; Apgar scores at 1, 5, and 10 minutes; resuscitation intensity described by the delivery room resuscitation description field grouped as follows from least to greatest intensity: 1) stimulation, dried, radiant warmer, bulb syringe; 2) CPAP, positive pressure ventilation, Oxygen saturation monitoring; 3) mask, T-piece; 4) “see code sheet”, epinephrine, chest compression, umbilical venous catheter, umbilical arterial catheter, intubation; chromosomal anomalies; congenital anomalies; severe birth trauma; maternal drug use; and fetal/placental conditions; The latter five conditions were defined as meeting criteria as defined in the Joint Commission “Unexpected complication in newborn” metric.[2] LightGBM, a gradient boosting algorithm, was used to model NICU admission and NICU days. LightGBM has outstanding

© 2020 Braun D et al. *JAMA Network Open.*

predictive performance while accommodating issues such as complex interactions of candidate factors, non-linearity and missing data.[3] . A logistic objective function was used to estimate adjusted NICU admission probability, and a Poisson objective function was used to estimate adjusted NICU patient days. The data were randomly split into a training set, representing 80% of the data, and a testing set, representing 20% of the data. Hyperparameters for the models were selected by performing Bayesian optimization on five-fold cross validated estimates of the models' performance on the training data.[4] All model performance statistics were calculated on the remaining testing data.

© 2020 Braun D et al. *JAMA Network Open.*

Model Covariates

Baby	Mother	Socio-Economic (Mother's Census Tract)	Joint Commission UNC Denominator Categories
Birth Weight	Delivery Type (Vaginal or C-Section)	Population Density	Congenital Malformation
Gestational Age	Cesarean Section Indication	Ethnic Population Proportions (Hispanic, African-American, Asian)	Fetal/Placental Conditions (excluding those related to birth weight/gestational age)
Kaiser Membership	Fetus Count	Education Population Proportions (High School, College, and Graduate)	Severe Birth Trauma
Medicaid Insurance	Gravidity	Median Residence Purchase Price	Maternal Drug Use
APGAR Scores (1, 5, 10 minute)	Perinatal care timeliness	Median Household Income	
Resuscitation Intensity	Magnesium Sulfate treatment	Unemployment Rate	
	Steroid treatment		
	Kaiser Membership		
	Medicaid Insurance		
	Ethnicity		

© 2020 Braun D et al. *JAMA Network Open.*

Model Diagnostics

Model Diagnostics: Neonatal Intensive Care Unit patient days (N = 39,366)

	Full	High GA/BW	Low GA/BW
R^2	0.729	0.233	0.606
RMSE (patient days)	10.712	7.312	19.81

Abbreviation: RMSE, Root Mean Square Error; High GA/BW, gestational age ≥ 35 weeks and birth weight ≥ 2000 g; Low GA/BW, gestational age < 35 weeks and birth weight < 2000 g (High GA/BW)

Model Diagnostics: Neonatal Intensive Care Unit Admission (N = 320,636)

	Full	High GA/BW	Low GA/BW
C-Statistic	0.895	0.849	0.949
Average Precision	0.74	0.529	0.995

Abbreviation: High GA/BW, gestational age ≥ 35 weeks and birth weight ≥ 2000 g; Low GA/BW, gestational age < 35 weeks and birth weight < 2000 g (High GA/BW)

Average Precision, defined as the area under precision-recall curve

© 2020 Braun D et al. *JAMA Network Open.*

eReferences

1. Chen W, Petitti DB, Enger S. Limitations and potential uses of census-based data on ethnicity in a diverse community. *Ann Epidemiol.* 2004;14:339-345.
2. Joint Commission Specifications Manual for Joint Commission National Quality Measures (v2018B). https://manual.jointcommission.org/releases/TJC2018B/MIF0393.html, accessed June 27, 2019
3. Ke G, Meng Q, Finley T, Wang T, Chen W, Ma W, Ye Q, T L. "LightGBM: A Highly Efficient Gradient Boosting Decision Tree". Advances in Neural Information Processing Systems 30 (NIPS 2017), pp. 3149-31.
4. Snoek J, Larochelle H, . ARP. Practical Bayesian optimization of machine learning algorithms. In Proceedings of the 25th International Conference on Neural Information Processing Systems - Volume 2 (NIPS 12), F. Pereira, C. J. C. Burges, L. Bottou, and Q. Weinberger (Eds.), Vol. 2. Curran Associates Inc., USA, 2951-2959. https://papers.nips.cc/paper/4522-practical-bayesian-optimization-of-machine-learning-algorithms.pdf (Accessed on October 30, 2019). *2012.*

© 2020 Braun D et al. *JAMA Network Open.*
